# Automatic Evaluation of Bone Age Using Hand Radiographs and Pancorporal Radiographs in Adolescent Idiopathic Scoliosis

**DOI:** 10.3390/diagnostics15040452

**Published:** 2025-02-13

**Authors:** Ifrah Andleeb, Bilal Zahid Hussain, Julie Joncas, Soraya Barchi, Marjolaine Roy-Beaudry, Stefan Parent, Guy Grimard, Hubert Labelle, Luc Duong

**Affiliations:** 1 Department of Software and IT Engineering, École de Technologie Supérieure, Montréal, QC H3C 1K3, Canada; ifrahzhcet@gmail.com; 2 Department of Electrical and Computer Engineering, Texas A&M University, College Station, TX 77840, USA; zahidhussain909@tamu.edu; 3 Department of Orthopedics, CHU Sainte-Justine, Montréal, QC H3T 1C5, Canada; julie.joncas.hsj@ssss.gouv.qc.ca (J.J.); soraya.barchi@umontreal.ca (S.B.); marjolaine.roy-beaudry.hsj@ssss.gouv.qc.ca (M.R.-B.); stefan.parent@umontreal.ca (S.P.); guy.grimard.hsj@ssss.gouv.qc.ca (G.G.); hubert.labelle@umontreal.ca (H.L.); 4 Department of Surgery, Université de Montréal, Montréal, QC H3T 1J4, Canada

**Keywords:** radiographs, adolescent idiopathic scoliosis, activation maps, transfer learning, DenseNet, MAE, boneage, RSNA

## Abstract

**Background/Objectives:** Adolescent idiopathic scoliosis (AIS) is a complex, three-dimensional spinal deformity that requires monitoring of skeletal maturity for effective management. Accurate bone age assessment is important for evaluating developmental progress in AIS. Traditional methods rely on ossification center observations, but recent advances in deep learning (DL) might pave the way for automatic grading of bone age. **Methods:** The goal of this research is to propose a new deep neural network (DNN) and evaluate class activation maps for bone age assessment in AIS using hand radiographs. We developed a custom neural network based on DenseNet201 and trained it on the RSNA Bone Age dataset. **Results:** The model achieves an average mean absolute error (MAE) of 4.87 months on more than 250 clinical testing AIS patient dataset. To enhance transparency and trust, we introduced Score-CAM, an explainability tool that reveals the regions of interest contributing to accurate bone age predictions. We compared our model with the BoneXpert system, demonstrating similar performance, which signifies the potential of our approach to reduce inter-rater variability and expedite clinical decision-making. **Conclusions:** This study outlines the role of deep learning in improving the precision and efficiency of bone age assessment, particularly for AIS patients. Future work involves the detection of other regions of interest and the integration of other ossification centers.

## 1. Introduction

Adolescent idiopathic scoliosis (AIS) is a characterized by a deformation of the spine in 3-D [1]. In AIS, observations of the illiac crests, namely, the Risser grade, are routinely used to assess the skeletal maturity [2]. This information is highly relevant to assess the peak growth rate velocity, the growth rate and the final adult height [3]. This information is crucial for the management of AIS and guiding treatment [4].

**Figure 1:**
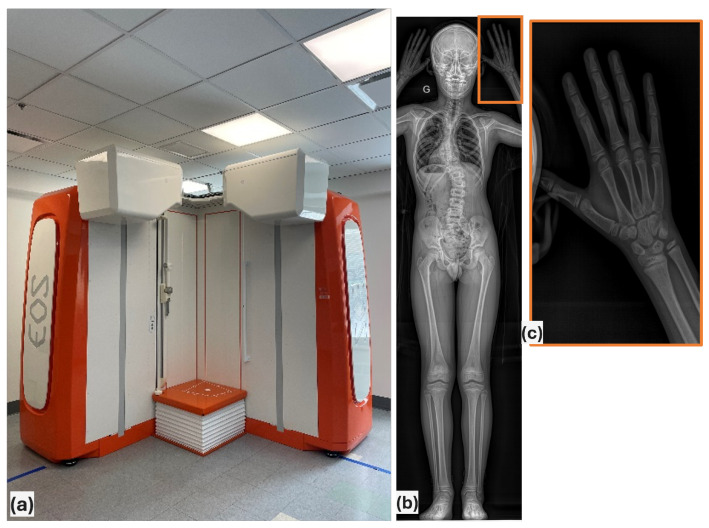
(**a**) EOS imaging system Pancorporal, (**b**) Radiograph of AIS patients, (**c**) Matching hand/wrist images [4].

Two common methods for assessing bone age are the Greulich-Pyle (GP) [5] and Tanner-Whitehouse (TW) [6] methods. In the GP method, doctors compare X-rays of the hand and wrist with a reference book of images to estimate bone age. The TW method is more detailed, in which clinicians score each bone in the hand and wrist, adding up the scores to determine bone age. For AIS patients, the Sanders classification is often used. This method is simpler than the TW method and was designed to be quick, reliable, and helpful for tracking the type and severity of the spinal curve [7]. Some examples of wrist radiographs of AIS patients are shown in Figure 1.

Lee et al. [8] evaluated 3000 hand images annotated with key feature points, enabling the precise selection of relevant regions for age estimation. Distinct regions of interest (ROIs) were defined, including small areas of carpal and metacarpal bones and larger sections encompassing phalanges. Histogram equalization was applied to minimize irrelevant intensity variations in cropped images. Addressing gender-based growth rate differences, separate models were developed. Notably, the research embraced all age ranges, including scarce data from infancy and early childhood, often overlooked due to morphological differences. By training various deep learning architectures with different ROI definitions, the study achieved a minimum mean absolute difference error of 8.890 months on a test set of 400 images. This preliminary investigation paves the way for future research into unexplored alternative approaches.

Kaddioui et al. [9], retrospectively collected and manually graded 1830 posteroanterior radiographs of adolescent patients with AIS using the United States Risser staging system. The radiographs underwent preprocessing and were cropped to focus on the pelvic region. A convolutional neural network (CNN) was trained to automate Risser classification, achieving accuracy comparable to human graders. The network’s performance was validated against interobserver variability using the Fleiss measure. Due to limited radiograph availability, transfer learning was employed with the VGG16 network. The agreement among six observers was moderate, with a coefficient of 0.65 and 74.5% concordance. The automated method achieved substantial agreement (=0.72) and an overall accuracy of 78.0% compared to ground truth. This research demonstrates the potential of deep learning in automating AIS grading, even with a limited dataset, and establishes a promising foundation for improving scoliosis assessment in clinical practice. Magnide et al. [10] have used a pre-trained ResNet101 model, with a SVM classifier to classify different region of interests on pancorporeal radiographs of AIS patients (pelvis, humeral heads, femoral heads) at 5 different visits. Visualisation of class activation maps (CAM) using gradient-weighted class activation maps (Grad-CAM) was used to outline the different ossification centers and validation for follow up. Unfortunately, the posture of AIS patient does not allow a good visualisation of the hand and a second radiograph of the hand/wrist was often required to assess precisely the bone age. Recently, we have introduced a new posture in pancorporeal radiographs, to include the wrist and hand.

Pan et al. [11] proposed a comprehensive evaluation of bone age assessment methods using deep learning models and expert radiologists. The study focuses on a Convolutional Neural Network (CNN) named TDL-BAAM, trained on 15,129 pediatric trauma hand radiographs from Children’s Hospital of New York. CNN’s predictive ability was compared against a GP-based deep learning model (GPDL-BAAM) and two pediatric radiologists using the GP method. An independent test set of 214 trauma hand radiographs from Hasbro Children’s Hospital was used for validation. The TDL-BAAM achieved a mean absolute error (MAE) of 11.1 months, outperforming GPDL-BAAM (12.9 months) and the radiologists (14.6 and 16.0 months). TDL-BAAM’s predictions were within 24 months of chronological age for 95.3% of cases, compared to 91.6% for GPDL- BAAM and lower percentages for radiologists. High concordance (Intraclass Correlation Correlation Coefficient ICC: 0.93) existed between all methods and chronological age. The deep learning models showed a systematic bias, leaning towards overestimating age for younger children, unlike radiologists who exhibited consistent biases. This study underscores the potential of deep learning for accurate bone age assessment, with TDL-BAAM demonstrating notable performance improvements over traditional methods and human experts.

In recent years, the recent advances in artificial intelligence, particularly deep learning, have found to be promising for medical image analysis [12,13]. Convolutional neural networks (CNNs) might contribute to reducing the reading time compared to methods. Despite this progress, the transition from research to clinical application necessitates rigorous validation and adaptation to real-world scenarios. The application of deep learning models in medical contexts demands external validation studies employing diverse datasets to ensure generalizability and robustness.

The goal of this study is to present a new bone age classification approach based on deep neural networks and on class activation maps for wrist radiographs from EOS radiographs. We evaluated our results on the Radiological Society of North America (RSNA) Bone Age dataset and on clinical EOS radiograph AIS patients.

## 2. Methodology

### 2.1. Dataset

**Figure 2:**
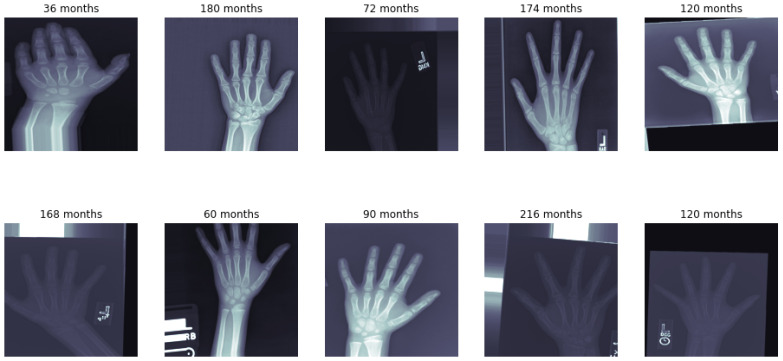
Sample hand and wrist radiograph from the RSNA Bone Age dataset.

Our model was trained on the RSNA Bone Age dataset. The RSNA Bone Age dataset [14] is a freely accessible set of data created to support the development and testing of automated skeletal age estimation techniques. It comprises 12,000 hand radiographs at different ages, from infants to late teens as depicted in Table 1. Both male and female participants are represented in the dataset. The radiographs were acquired from several hospitals and imaging facilities. Some sample images from the dataset are shown in Figure 2.

The proposed model was evaluated on a cohort of 257 clinical AIS patients. First, the region of interest of the hand and wrist was manually extracted from full-standing EOS radiographs using GNU image processing software (GIMP) which is free and open-source. Then, the bone age of AIS patient was evaluated by using BoneXpert (Visiana ApS, Hørsholm, DEN) [15]. BoneXpert, employs advanced active appearance models (AAMs) and machine learning techniques to automate bone age estimation [16], with a reported accuracy of 95%. By reconstructing bone borders, computing intrinsic bone ages based on shape, intensity, and texture scores, and subsequently transforming them into GP or TW bone ages.

**Figure 3:**
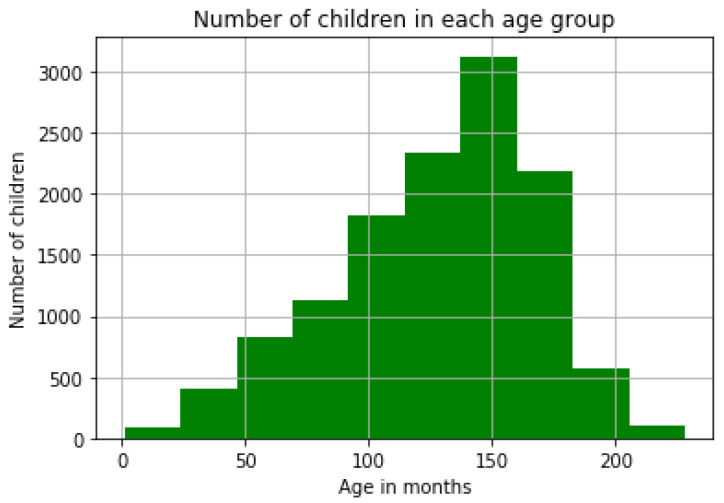
Distribution of bone age of the children in months.

The skeletal age of the patient was obtained by a corresponding skeletal age label that is present with every hand radiograph in the dataset. The maturation of certain bones is evaluated, and their comparison to accepted reference atlases yields the bone age. The dataset includes information about the patient’s age, gender, and hand side (left or right), in addition to the hand radiographs and bone age labels. The bone age distribution of various patients are shown in Figure 3.

In terms of dataset utilization for model training and testing, a split ratio of 80:20 is employed. This allocation ensures that 80% of the data is used for training the model, allowing it to learn and adapt to various patterns and nuances present in the radiographs. The remaining 20% of the data is reserved for testing, providing a robust means to evaluate the model’s effectiveness and accuracy in bone age estimation.

### 2.2. Initial Experiments

The initial exploration in bone age estimation involved conducting experiments with three transfer learning models: Visual Geometry Group (VGG16 and VGG19) (University of Oxford, Department of Engineering Science, Oxford, UK) and Inception (Google AI, Google LLC., Mountain View, CA, USA). The relatively high MAE values for each model underscored their limitations. The experiments with VGG16, VGG19, and Inception models were conducted under uniform hyperparameters: a batch size of 32, the Rectified Linear Unit (ReLU) activation function, and the Adam optimizer. VGG16 consists of 16 layers, VGG19 has 19 layers, and Inception involves a more intricate architecture with multiple convolution layers. Despite this, their performance was suboptimal, as indicated by high MAE values. This could be attributed to their architectures being less suited for the specific nuances of bone age imaging, possibly lacking in capturing fine-grained details critical for accurate age estimation. These preliminary trials were essential in guiding the direction of our research.

### 2.3. Experimental Design

**Figure 4:**
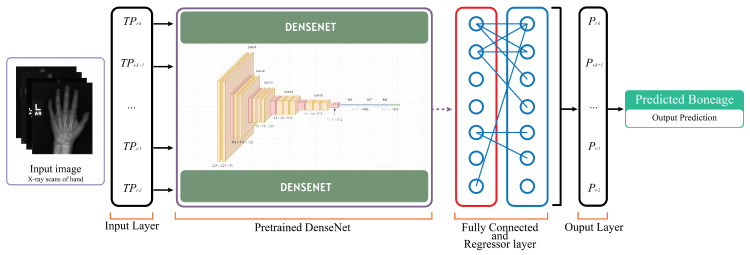
Deep Learning Model Architecture for BoneAge Prediction.

The bone age estimation model uses X-ray images of wrists as input to the proposed deep learning (DL) model. The model employs a fine-tuned transfer learning approach with DenseNet201 (Linux Foundation, San Francisco, CA, USA) for maximum accuracy and minimal MAE and leverages DenseNet201’s pre-trained features for efficient feature extraction. DenseNet201 is fine-tuned by taking it as a base layer and then integrating our own uniquely designed CNN layers. This approach significantly reduces the computational cost and training time required while still delivering good accuracy. The proposed experimental bone age estimation neural network architecture is shown in Figure 4.

### 2.4. Network Architecture

Each image from the RSNA bone age dataset undergoes preprocessing, including resizing to 299 × 299, to conserve computational resources. This resizing step was crucial for speeding up the processing and minimizing memory consumption. The first layer of our architecture consists of “Densenet201” as a functional transfer learning layer that takes the input image and performs convolutional operations to extract features. The architecture of DenseNet201 consists of multiple densely connected blocks, each containing several layers. Unlike traditional CNNs, DenseNet201 employs skip connections, or shortcuts, that connect every layer to every other layer within the same block. This dense connectivity pattern not only facilitates feature reuse but also addresses the vanishing gradient problem commonly encountered in deep networks. The Densenet201 instantiation notably omits the fully connected layer and integrates pre-trained weights from the ImageNet (Stanford Vision Lab, Stanford University, Stanford, CA, USA) database, a standard practice in transfer learning to leverage pre-learned feature mappings. The “Densenet201” layer gives an output shape of (None, 9, 9, 1920), indicating that the input image is transformed into a feature map with a size of 9 × 9 and 1920 channels.

**Figure 5:**
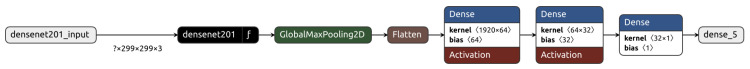
DenseNet201 Model Architecture for BoneAge Prediction.

Once the DenseNet201 architecture has processed the input, its output serves as the input for our custom-designed CNN layers. Our CNN architecture is designed to better recognize and understand the detailed patterns in the X-ray images. Figure 5 shows the detailed description of the DL model used for bone age prediction.

Subsequent to the integration of DenseNet201, the proposed custom CNN employs a GlobalMaxPooling2D layer. This layer effectively reduces the dimensions of the input and concentrates on the important features required for bone age estimation. This reduction is followed by a Flatten layer, which transforms the condensed feature maps into a linear array. The model further consists of a sequence of three dense layers. The first and second of these layers comprise 64 and 32 neurons, respectively, and employ the ‘relu’ activation function. This function is instrumental in introducing non-linear characteristics to the model, enabling it to decipher more intricate patterns in the data. The final layer in this sequence is a single neuron dense layer utilizing a ‘linear’ activation function, implying that the model’s output is a singular continuous variable such as the bone age.

Regarding optimization techniques, multiple optimizers are defined: stochastic Gradient descent (SGD), nesterov-accelerated adaptive moment estimation (Nadam), and Adamax. However, the compilation of the proposed custom CNN is exclusively with the Adamax optimizer. This optimizer is configured with a learning rate of 0.001, and the model employs a mean squared error (MSE) loss function, which aligns with regression-based objectives. The primary metric for evaluating model performance is articulated as ‘mae_in_months’, representing mean absolute error in months, a metric particularly pertinent in applications of precise continuous bone age value predictions. In terms of the number of parameters, the model has 18,447,041 parameters with a total batch size of 32. Among them, 18,217,985 parameters are trainable, meaning they are updated during the training process to optimize the model’s performance. The remaining 229,056 parameters are non-trainable, representing fixed weights that are not updated.

### 2.5. Model Explainability: Score-CAM

CAMs also called heatmaps, are a way to understand how CNNs make their predictions. In this study, we used a method called score-weighted class activation maps (Score-CAM), which measures the importance of different parts of an image by looking at how input features affect the output. Unlike traditional methods that rely on gradients (a kind of sensitivity measurement), Score-CAM takes a broader view of the input’s impact. It [17] is also useful for identifying issues in the model, such as why it might make an incorrect prediction or if there are biases in the dataset. This makes it a powerful tool for improving and understanding deep learning models.

The reason for choosing Score-CAM [18] lies in the fact that it typically outperforms other methods across a wide range of metrics, with the exception of complexity. Furthermore, in [18], it’s worth mentioning that Score-CAM shows strong performance with VGG and ResNet architectures. Conversely, Grad-CAM [19] scores the highest in the ADCC score with ResNeXt models.

### 2.6. Evaluation Metrics

Mean absolute error (mae_in_months): MAE [20], generally expressed in months for bone age, is a measurement of the average size of errors in a set of predictions. It is used to evaluate the efficacy of a regression model and is quantified as the average absolute difference between the predicted values and the actual values. The MAE loss function formula:

(1) $$\mathrm{MAE}=\frac{1}{n}\sum_{i=1}^{n}\left|y_{i}-{\hat{y}}_{i}\right|$$

where:

*n* is the number of observations in the dataset,

$y_{i}$ is the true value,

${\hat{y}}_{i}$ is the predicted value

“MAE months” calculates MAE in months, thereby quantifying the discrepancy between anticipated bone ages and factual bone ages. This metric has significant medical relevance, facilitating an intelligible measure of the model’s precision in bone age estimation. This function, characterized by two inputs, normalized predicted bone ages and normalized actual bone ages transforms these values back to their original scale using calculated mean and standard deviation from the training dataset. This transformation yields an MAE in months, a temporal dimension of considerable clinical significance. A decreased MAE signifies better alignment between projections and factual bone ages.

## 3. Results

The proposed neural network, trained for 35 epochs, proposes a solution of automating bone age assessment using machine learning. Furthermore, we explored the benefits of using an automated boneage prediction method using DL, which leads to improved MAE, accuracy, efficiency, and decision making for support in managing scoliosis patients.

**Figure 6:**
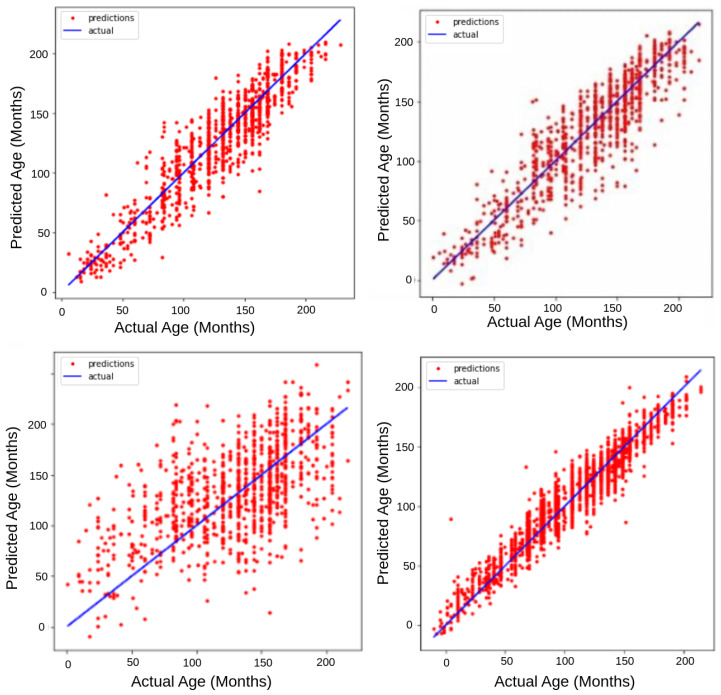
Graphical Comparison of VGG16, VGG19, Inception and Proposed Model trained on the same dataset.

To assess the effectiveness of various models in bone age estimation, initially several experiments with VGG16, VGG19, and Inception, were done while ensuring consistency in hyperparameters across all trials. A detailed evaluation of the performance of each model was done, where VGG16 showed a MAE of 11.35 months, VGG19 had an MAE of 14.34 months, and Inception yielded an MAE of 32.5 months. Figure 6 presents a detailed graphical comparison of the performance metrics in these different models.

In a scatter plot as shown in Figure 6, the model’s accuracy is inferred by how closely the data points cluster around the line representing the actual age. The VGG16 model on the top left plots a noticeable improvement in prediction accuracy. The points are more tightly clustered around the line, especially in the mid-range of ages. However, some discrepancies remain for younger and older ages. Moving to the top right VGG19 plot, it depicts a model with significant variance, as the data points are quite spread out from the actual age line. This model tends to underpredict ages for younger subjects and overpredict for older ones, which is suggested by the curvature of the data points away from the actual age line. The bottom left Inception plot displays the weakest predictive performance among the four. The data points show a considerable spread, indicating high MAE, and there is a consistent trend where the model underpredicts the actual age across the full age range. In contrast, the bottom right proposed model plot showcases the most accurate model. The predictions are closely aligned with the actual ages, with data points densely packed around the line, showing low MAE values and minimal bias. Overall, the proposed model is the most precise, while the Inception model significantly underperforms. The resulting MAE of 4.87 months and an $R^{2}$ value of 0.975 achieved by our custom network demonstrates the effectiveness of the applied approach and justifies the decision to invest in the development of a specialized model.

**Figure 7:**
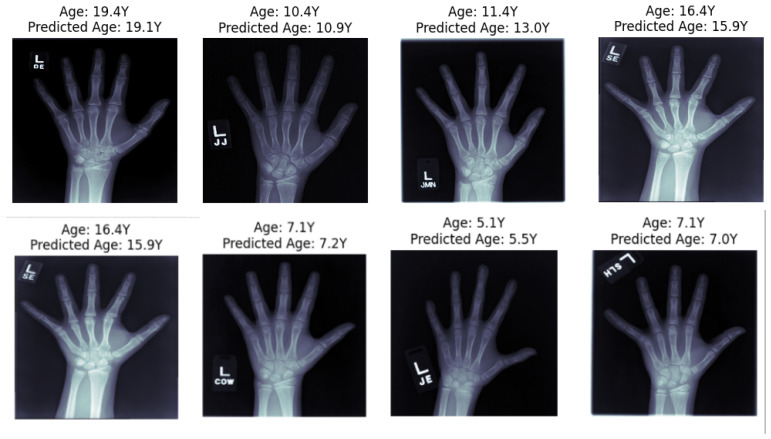
Actual Age versus Predicted Age results.

The predicted bone age values generated by our custom neural network were compared with the actual bone age labels for the hand radiographs in Figure 7. It indicates that the proposed model generally predicts with a high degree of accuracy, as evident by the close proximity of the predicted and actual ages in several instances, such as a predicted age of 7.1 years being near an actual age of 7.2 years. However, there are some instances where the proposed model’s predictions deviate from the actual predictions, as seen in an outlier where the model predicted 13.0 years compared to the actual age of 11.4 years, this can be attributed to various factors related to the inconsistencies in image quality such as contrast, brightness, and noise levels. Furthermore, the model might struggle with atypical bone structures or rare pathologies that are underrepresented in the training set, leading to inaccurate predictions for such cases.

**Figure 8:**
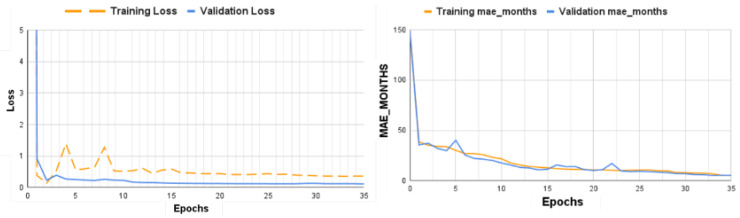
Loss and MAE month curve plots for proposed model.

The results show a similarity between the predicted and actual bone ages. The small difference between the predicted and actual values further validates the effectiveness of our approach in accurately assessing bone age in hand radiographs. Figure 8 illustrates the model’s loss graph, depicting its training progress and validation process, and also displays the MAE Month graph, measuring the training and validation mae_in_months values. Using automation and advanced algorithms, our approach enhances the diagnostic process and facilitates personalized treatment planning, ultimately resulting in better patient outcomes. Table 2 presents a comparison of the performance of the proposed bone age CNN model with different models from the literature.

**Figure 9:**
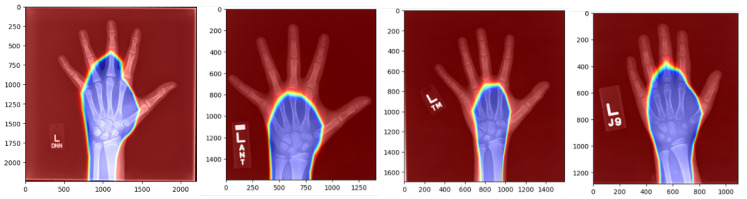
Score-CAM Results of the proposed neural network, showing its attention which highlights the important areas found in the images for determining boneage. In this color map, blue shows the most significant areas, and red shows the least significant ones.

In addition, we utilized the Score-CAM model as saliency maps of Score-CAM are more focused than Grad-CAM [17]. Score-CAM was used to generate visual maps that evaluate the performance of our proposed method, which visually represents the areas in the wrist bone images that contribute most significantly to accurate bone age in months. The results of the feature activation heat map analysis are depicted in Figure 9, illustrating the effectiveness of our approach in identifying and highlighting the key bone regions. These heat maps provide valuable insights into the regions that play a crucial role in determining bone age and aid in understanding the decision-making process of our deep neural network. This process enhances the robustness of our method without requiring additional segmentation steps, reducing computational complexity and improving efficiency. The Score-CAM image provides a visual representation of how the neural network processes data for bone age estimation in AIS patients. The areas where the network concentrates its attention are distinctly marked in blue, indicating regions of high focus. In contrast, the areas shown in red represent regions where the network’s focus is significantly lower.

**Figure 10:**
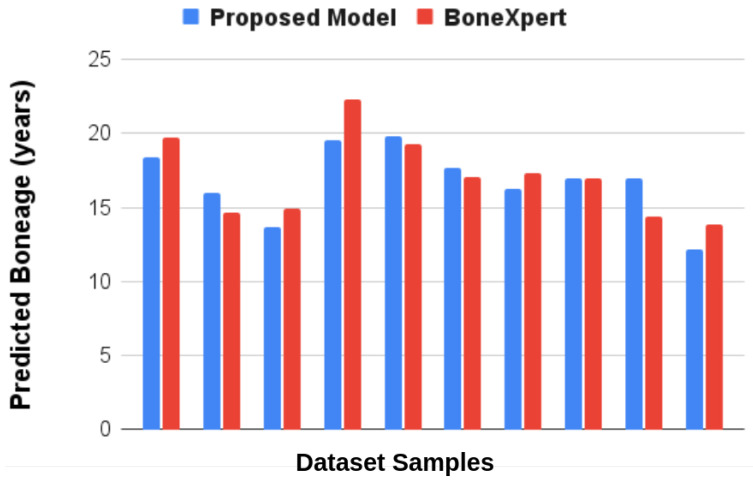
Comparison of the performance of the Proposed Model with BoneXpert.

The mae_in_months was used as the evaluation metric, offering a quantitative measure of the predictive models’ performance in estimating bone age accurately. The results of the proposed method as compared to the performance of BoneXpert are shown in Figure 10. The bar chart compares the predicted bone age from two different models: the proposed model and BoneXpert, across various age groups. Both models yield similar results, with slight variations in predictions. The similarity between the mae_in_months values of both the BoneXpert system and the proposed research model underlines their comparable performance in predicting bone age.

## 4. Discussion

The primary question this research aims to address is whether a custom CNN can reliably and accurately assess bone age in AIS patients using hand radiographs. This is particularly important for evaluating skeletal maturity, which is a critical factor in managing AIS progression and treatment planning. The study also examines the potential of explainability tools, such as Score-CAM, to improve the transparency of the model in clinical settings. The proposed model demonstrates promising results in predicting bone age, closely matching BoneXpert in several instances. For example, the proposed model’s prediction is close to BoneXpert’s. Furthermore, when the proposed model predicts lower than BoneXpert, like the 15.95 versus 14.62 years, it suggests the model could be more sensitive in detecting earlier maturation stages, which can be crucial for early intervention strategies. Even though this is a preliminary study, the results indicate a good correlation with the BoneXpert model.

The paper’s methodology section outlines the development of a custom neural network (NN) architecture based on DenseNet201, leveraging transfer learning to achieve low MAE for AIS patients is novel. Unlike many general bone age assessment studies, this work specifically addresses AIS, a niche yet clinically significant subgroup. This approach not only reduces computational complexity but also yields a MAE of 4.87 months, surpassing other pre-trained models like VGG16 and VGG19, demonstrating the effectiveness of domain-specific model development. Furthermore, the paper introduces Score-CAM as an explainability tool, shedding light on the regions of interest within hand radiographs that contribute most significantly to accurate bone age estimation. This visual explanation enhances the trust and transparency of the DNN model, making it more acceptable in clinical practice. Introducing Score-CAM to visualize the regions of interest in the radiographs highlights a unique aspect of this study, ensuring clinical relevance and interpretability.

The study compares the proposed DNN’s performance with the BoneXpert system, a leading approach in the field. The use of automated techniques to evaluate the bone age might be highly relevant in the study of spinal deformities. Our results reveal similar MAE values, showcasing the potential of the proposed DNN to be a reliable and efficient tool for bone age prediction. This convergence in performance signifies the progress in automating bone age assessment, reducing inter-rater variability, and expediting clinical decision-making. The ability of the proposed method to extract key areas of the bone in an explainable way is a significant advantage. By gaining insight into which regions are influential in the classification process, clinicians and radiologists can better understand the algorithm’s decision and have greater confidence in its results. The explainability aspect is particularly important in medical applications as it enables trust, transparency, and acceptance of the automated system. This study addresses a critical gap in the field: the need for automated and interpretable tools for bone age assessment in AIS patients. Current approaches often rely on subjective manual assessments or black-box AI models with limited clinical adoption due to a lack of transparency. By combining automation with interpretability, the proposed approach aims to bridge the gap between technological advances and clinical usability.

The proposed methodology also has some limitations which could be improved in the future works. Our method requires to outline manually the region of interest of the hand from the pancorporal radiograph. This step can be automated using a recent object detection model such as Masked R-CNN, but it remains to be evaluated. The proposed bone age estimation model may exhibit biases due to data imbalances and lacks representation of diverse boneage demographics. Its accuracy heavily depends on the input X-ray image quality and varies with equipment differences. Additionally, the model’s reliability in rare conditions or anomalies affecting bone development, not well-represented in the training data, is uncertain. Furthermore, the use of DenseNet201, known for its densely connected architecture, along with additional custom layers, could lead to a computationally intensive model. This might require significant computational resources for training and inference, potentially limiting its use in environments with limited processing capabilities. Furthermore, researchers could explore the integration of advanced object detection models, such as Masked R-CNN or YOLO, to automate the extraction of the hand region from pancorporal radiographs. This will not only streamline the workflow, but also reduce potential human error and improve reproducibility. Future models should be trained with additional data that include rare anomalies that affect bone development. Collaboration with specialized medical institutions could provide access to such datasets, enhancing applicability of the model in less common clinical scenarios.

## 5. Conclusions

This study presents a deep neural network-based approach for pediatric bone age assessment, demonstrating its potential for the field of pediatric radiology in general, but also applied to AIS patients. The custom DNN architecture, combined with transfer learning and Score-CAM for explainability, results in accurate and transparent bone age predictions. To further enhance the model’s accuracy and robustness, collecting and annotating a more extensive and diverse dataset of pediatric hand radiographs, encompassing various age groups, ethnicities, and clinical conditions, would be beneficial. Data augmentation techniques can also be employed to artificially increase the dataset size. At the moment, the identification of the region of interest of the hand and wrist is manual, but future work will involve the automatic identification of the region of interest and the evaluation of BoneXpert on a larger amount of AIS patients. The adoption of deep neural networks in bone age assessment holds promise for better monitoring of growth progression in pediatric populations.

## Figures and Tables

**Table 1:** Patient Demographics from RSNA Bone Age Dataset.

Metric	Value
Total Patients	12,611
Male Patients	6833
Female Patients	5778
Mean Age	10.61
0–5	801
6–10	3487
11–15	7224
16–20	1099

**Table 2:** Comparison of the performance of models.

Model	Dataset	MAE (Months)
Kim et al., 2023 [21]	Private Data	10.5
Nam et al., 2023 [22]	RSNA Bone Age	7.43
Toka et al., 2023 [23]	RSNA Bone Age	6.32
Guo et al., 2022 [24]	RSNA Bone Age	6.07
Moszczynska et al., 2020 [25]	RSNA Bone Age	8.18
Mahayossanunt et al., 2019 [26]	RSNA Bone Age	6.20
**Proposed Model**	RSNA Bone Age	**4.87**

## Data Availability

Publicly available dataset was analyzed in this study. This data can be found here: https://www.rsna.org/rsnai/ai-image-challenge/rsna-pediatric-bone-age-challenge-2017 (accessed on 20 December 2024).

## Abbreviations

The following abbreviations are used in this manuscript:

AIS	Adolescent Idiopathic Scoliosis
CNN	Convolutional Neural Network
CAM	Class Activation Map
GP	Greulich-Pyle
TW	Tanner-Whitehouse
RSNA	Radiological Society of North America
MAE	Mean Absolute Error
ROI	Region of Interest
GPDL-BAAM	Greulich-Pyle Deep Learning Bone Age Assessment Model
TDL-BAAM	Tanner-Whitehouse Deep Learning Bone Age Assessment Model
ICC	Intraclass Correlation Coefficient
VGG	Visual Geometry Group
Inception	Deep Learning Model Architecture (GoogleNet)
DenseNet201	Dense Convolutional Network with 201 Layers
ReLU	Rectified Linear Unit
MSE	Mean Squared Error
SVM	Support Vector Machine
Score-CAM	Score-Weighted Class Activation Map
Grad-CAM	Gradient-Weighted Class Activation Map
EOS	End of Scoliosis
AAM	Active Appearance Model
BoneXpert	Commercial Bone Age Grading Software
GIMP	GNU Image Manipulation Program
